# The relationship between outpatient service use and emergency department visits among people treated for mental and substance use disorders: analysis of population-based administrative data in British Columbia, Canada

**DOI:** 10.1186/s12913-022-07759-z

**Published:** 2022-04-11

**Authors:** M. Ruth Lavergne, Jackson P. Loyal, Mehdi Shirmaleki, Ridhwana Kaoser, Tonia Nicholls, Christian G. Schütz, Adam Vaughan, Hasina Samji, Joseph H. Puyat, Megan Kaulius, Wayne Jones, William Small

**Affiliations:** 1 Department of Family Medicine, Faculty of Medicine, Primary Care Research Unit, Dalhousie University, 1465 Brenton Street, Suite 402, Halifax, NS B3J 3T4 Canada; 2 Centre for Applied Research in Mental Health and Addiction, Simon Fraser University, Vancouver, BC Canada; 3 BC Centre for Disease Control, Vancouver, BC Canada; 4 Department of Psychiatry, University of British Columbia, Vancouver, BC Canada; 5 Department of Psychology, Simon Fraser University, Burnaby, BC Canada; 6 BC Mental Health and Substance Use Services, Vancouver, BC Canada; 7 Centre for Health Evaluation and Outcome Sciences, University of British Columbia, Vancouver, BC Canada; 8 School of Criminal Justice and Criminology, Texas State University, San Marcos, TX Canada; 9 Faculty of Health Sciences, Simon Fraser University, Burnaby, BC Canada; 10 School of Population and Public Health, University of British Columbia, Vancouver, BC Canada; 11 BC Centre on Substance Use, Vancouver, BC Canada

**Keywords:** Mental disorders, Substance use disorders, Ambulatory care, Primary care, Continuity of care, Emergency services, Emergency department visits, Administrative data

## Abstract

**Background:**

Research findings on the association between outpatient service use and emergency department (ED) visits for mental and substance use disorders (MSUDs) are mixed and may differ by disorder type.

**Methods:**

We used population-based linked administrative data in British Columbia, Canada to examine associations between outpatient primary care and psychiatry service use and ED visits among people ages 15 and older, comparing across people treated for three disorder categories: common mental disorders (MDs) (depressive, anxiety, and/or post-traumatic stress disorders), serious MDs (schizophrenia spectrum and/or bipolar disorders), and substance use disorders (SUDs) in 2016/7. We used hurdle models to examine the association between outpatient service use and odds of any ED visit for MSUDs as well count of ED visits for MSUDs, stratified by cohort in 2017/8.

**Results:**

Having had one or more MSUD-related primary care visit was associated with lower odds of any ED visit among people treated for common MDs and SUDs but not people treated for serious MDs. Continuity of primary care was associated with slightly lower ED use in all cohorts. One or more outpatient psychiatrist visits was associated with lower odds of ED visits among people treated for serious MDs and SUDs, but not among people with common MDs.

**Conclusion:**

Findings highlight the importance of expanded access to outpatient specialist mental health services, particularly for people with serious MDs and SUDs, and collaborative models that can support primary care providers treating people with MSUDs.

**Supplementary Information:**

The online version contains supplementary material available at 10.1186/s12913-022-07759-z.

## Background

Emergency departments (EDs) provide timely access to acute care for mental and substance use disorders (MSUDs) and fill gaps for people who cannot otherwise access services [1–3]. On the other hand, ED visits for MSUDs may suggest that peoples’ conditions were poorly managed in the community, that they had difficulty accessing outpatient primary care and/or psychiatry services when they needed them [1, 4–6]. Improving appropriate use of EDs for MSUDs is a topic of ongoing policy consideration in many jurisdictions, particularly as people visiting the ED for MSUDs treatment often present frequently [5, 7, 8]. Information about the relationship between outpatient service use and ED visits is essential to inform policy and system planning.

While there is a large body of literature examining factors shaping ED service use generally [9–12], research on factors shaping ED visits for MSUDs is limited and findings are mixed. Studies have found that primary care and outpatient psychiatric care use is associated with greater [5, 7, 8, 13–20], lower [14, 15, 20–25], or no difference [17, 18] in ED utilization. These inconsistent findings demonstrate the need for further research, and in particular studies that account for disorder type as associations may differ between disorders [5, 14, 15]. Additionally, much of the existing research focuses on outpatient service use among ED users, excluding people who only use outpatient services. There is a consequent gap in information on the relationship between outpatient service use and *any* subsequent ED visits, in addition to count or frequency of visits.

We used province-wide population-based linked data in British Columbia (BC), Canada to examine the associations between outpatient service use (all primary care visits, primary care visits for MSUDs, continuity of primary care, outpatient psychiatry visits) and ED visits. We compared results between people treated only for common MDs (depressive disorders and/or anxiety disorders and/or posttraumatic stress disorder (PTSD)), people treated for serious MDs (schizophrenia spectrum and/or bipolar disorders, alone or concurrently with common MDs), and people treated for substance use disorders (SUDs) (including alcohol-related disorders, opioid-related disorders, cannabis-related disorders, stimulant-related disorders, and other substance use/abuse, alone or concurrently with common and/or serious MDs). We model both odds of any ED service use as well as count of ED visits.

## Methods

### Study setting

BC is the westernmost province in Canada and has a population of approximately 5 million people as of 2020. The province is organized into five regional Health Authorities which are responsible for the organization and delivery of hospital and ED services within their respective geographic regions. The provincial health insurance program (Medical Services Plan or MSP) covers all permanent residents, except for approximately 4% of the population covered under federal health insurance programs (including members of the Canadian Armed Forces). BC residents insured under MSP receive first-dollar coverage for all medically necessary services provided by licensed physicians or in hospital. Primary care plays a gatekeeping role, which means access to psychiatrists and other specialists requires a referral from primary care. Primary care is provided mostly by family physicians in private, fee-for-service family practices and walk-in clinics. Outpatient psychiatry is similarly reimbursed on a fee-for-service basis, with alternate payments predominantly for non-clinical duties [26]. It is individual physicians’ responsibility to arrange coverage for after-hours or weekend care, and patients may be directed to the ED if services are needed outside of practice hours.

### Data

We used individual-level de-identified data holdings from the BC Ministry of Health that are linked using both deterministic and probabilistic approaches and made accessible through Population Data BC [27]. Data included all people ages 15+ who are insured under the provincial insurance plan and who were treated for MSUDs. We used patient registry information to define the study cohort and to describe individual characteristics such as age, sex/gender, and location of residence [28]. ED visits were captured using the National Ambulatory Care Reporting System (NACRS) [29] and MSP physician billing data [30]. Physician service use was captured using billing data as well, and hospitalizations were identified using the Discharge Abstract Database [31]. Vital statistics deaths data was used to identify and exclude people who died during the study period [32].

### Study population

The study population includes all BC residents aged 15 years and older who were alive and registered for provincial health insurance between April 1 2016 and March 31 2018, and who had two or more outpatient visits, and/or one hospitalization for MSUDs (Appendix Table 4) in 2016/7. We excluded people with missing data for age or sex/gender.

### Measures

#### Mental and substance use disorders

We grouped people into three cohorts considering prevalence, typical severity of disorders, and treatment. Common disorders included depressive disorders, anxiety disorders, and PTSD. These are more prevalent, on average less severe, and frequently are managed in primary care settings [33–36]. Serious mental disorders included schizophrenia spectrum and bipolar disorders. These are less prevalent [37, 38], often more complex, persistent, associated with severe functional impairment, and typically require specialty education and resources to provide care [21, 39–42]. SUDs comprised the final cohort. In BC SUDs are frequently treated by primary care providers, but generally require different treatments to MDs especially in relation to the physical sequalae of substance use. People treated for both a common MD and a serious MD were included in the serious MDs cohort. People treated for both a MD (common and/or serious) and a SUD were grouped into the SUDs cohort.

#### Outpatient service use and continuity of care

We determined which people had an outpatient primary care visit for MSUDs and also counted the number of primary care visits (grouped into 0, 1, 2 or 3+ visits). Among people with substance use disorders, we counted the number of visits related to opioid agonist therapy separately, as these may include frequent visits to primary care providers who are not providing other primary care services. We calculated continuity of care with primary care physicians over this period using the Continuity of Care Index (COCI) [43]. The COCI identifies the number of primary care physicians providing services to a patient and the percentage of care provided by each physician. We scaled this to range from 0 (no visit/all visits to different physicians) to 10 (all visits with one physician) for clearer interpretation of regression parameters. People with only 1 primary care visit would have a value of 10. For this reason we included categories for people with 1 or 2 primary care visits in models as adjustment variables but focus our interpretation on parameter estimates for 3+ visits. We also examined psychiatrist outpatient visits (excluding visits with a hospital, day surgery, or ED service location code). For all patients seen in the ED in 2017/8, we examined outpatient service use in the 365 days preceding their first ED visit in 2017/8. For patients with no ED visits, we examined service use in the 2016/7 fiscal year.

#### Individual characteristics

Age was obtained from BC’s MSP registration file. Sex/gender is collected at time of MSP registration. The field is labeled “Gender” on the registration form but only the options “M” and “F” are provided. It is not possible to distinguish sex at birth, legal sex, and gender, based on this information, so we labelled this variable “sex/gender.” Health Authority was determined based on patient residential address, not location of service use. Neighbourhood income quintile was determined based on 2016 census enumeration area of residence, assigned using postal code conversion file developed and maintained by Statistics Canada [44, 45]. We used the Charlson-Deyo Comorbidity Index to measure multimorbidity. This index categorizes diagnosis codes based on 17 weighted categories [46, 47]. We identified people with hospitalizations for MSUDs, including involuntary hospitalizations under BC’s Mental Health Act in 2016/7 as a proxy indicator of severity.

#### MD ED visits (outcome)

Two data sources capture ED visits in BC: NACRS [29] and MSP payment information [30]. NACRS was developed by the Canadian Institute of Health Information to collect data on ED and other ambulatory visits. This captured 73% of ED visits in BC in 2017/8 as not all EDs submit data. All physician services delivered in EDs not reporting to NACRS are captured within the MSP billing data.

We identified MSP claims with a service location in the ED or corresponding to fee items billed only in the ED (Appendix Table 5). We also extracted all ED visits to BC facilities recorded in NACRS data. To ensure visits were not double counted across sources or when multiple MSP claims were submitted for a single patient, we retained only one ED record per patient, per day. Where multiple records contained different diagnoses, we retained records for MSUDs. We counted ED visits for MDs (including substance use) in 2017/8 as our outcome variable. We also report the number of ED visits for all other reasons in 2017/8.

### Analyses

We report descriptive statistics for individual characteristics and health services use for the three cohorts. We report treatment for MSUDs in 2017/8 by disorder group. Patients with two outpatient visits or one hospitalization (within a 365-day period) for the disorders listed in Appendix Table 4 were considered to have been treated for the disorder [48]. We used hurdle regression, with a logistic model (does a person have any vs no MSUD ED visits), and a left-truncated negative binomial model of the number of visits (among people with one or more visits) [11]. We report both unadjusted and adjusted results for service use variables. Adjusted models include all outpatient service use variables simultaneously, as well as sex/gender, age, health authority, rurality, income, MSUD diagnosis, Charlson-Deyo index, as well as binary flags for previous hospital admissions (all MSUD and involuntary) as indicators of severity. These variables are thought to be associated with outpatient service use and may also shape ED use [5, 8, 11, 15] and so we focus on adjusted odds ratios and rate ratios in interpretation to reflect the least biased estimates of the association between outpatient service use and ED visits.

### Ethics approval

This study was approved by the University of British Columbia, Providence Health Care Research Institute, and Simon Fraser University research ethics boards (REB number H17–00506). All inferences, opinions and conclusions drawn in this article are those of the authors, and do not reflect the opinions or policies of the data stewards.

## Results

We identified 336,973 people who were treated for MSUDs in 2016/7 and who were alive and registered throughout the study period, with final cohorts of 241,177 people treated for common MDs, 48,138 people treated for serious MDs, and 47,658 treated for SUDs (Table 1). Fewer than five people were excluded because of missing demographic data in each cohort. Among people treated for serious MDs, 27,135 (48.2%) were also treated for common MDs. Among people treated for SUDs, 21,866 (45.9%) were also treated for a common MD and 8109 (17.0%) were also treated for a serious MD. Among people treated for common MDs 31.8% were male, while 43.7% of people treated for serious MDs, and 62.9% of people treated for SUDs were male. Higher percentages of people treated for serious MDs were aged 45 years and older and a higher relative percentage lived in Vancouver Coastal health authority. Greater percentages of people treated for serious MDs and SUDS lived in lower income neighbourhoods and had prescriptions drug coverage tied to income assistance than people treated for common MDs. The Charlson-Deyo index for physical comorbidities was 0.8 among people treated for common MDs, 0.9 among people treated for serious MDs, and 1.0 among people treated for SUDs. Respective rates for total MSUD and involuntary hospital admission were 3.0 and 1.2% for common MDs, 19.5 and 13.2% for serious MDs, and 34.2 and 16.4% for SUDs (Table 1).

**Table 1:** Individual characteristics of patients treated for mental disorders (MDs) in 2016/7, by cohort. N(%) except where indicated

	Common mental disorders		Serious mental disorders		Substance use disorders	
	241,177	(71.6%)	48,138	(14.3%)	47,658	(14.1%)
Concurrent disorders						
Also treated for common MD	N/A	N/A	27,135	(48.2%)	21,866	(45.9%)
Also treated for serious MD	N/A	N/A	N/A	N/A	8109	(17.0%)
Sex/gender						
Female	164,373	(68.2%)	27,109	(56.3%)	17,666	(37.1%)
Male	76,804	(31.8%)	21,029	(43.7%)	29,992	(62.9%)
**Age**						
15–24	28,150	(11.7%)	4587	(9.5%)	4665	(9.8%)
25–44	78,793	(32.7%)	14,763	(30.7%)	20,844	(43.7%)
45–64	88,587	(36.7%)	19,618	(40.8%)	17,824	(37.4%)
65+	45,647	(18.9%)	9170	(19.0%)	4325	(9.1%)
Health Authority						
Interior	44,561	(18.5%)	6159	(12.8%)	8283	(17.4%)
Fraser	85,244	(35.3%)	17,830	(37.0%)	16,184	(34.0%)
Vancouver Coastal	51,243	(21.2%)	12,622	(26.2%)	11,160	(23.4%)
Vancouver Island	48,000	(19.9%)	8211	(17.1%)	8889	(18.7%)
Northern	11,969	(5.0%)	3241	(6.7%)	2442	(5.1%)
Unknown	160	(0.1%)	75	(0.2%)	700	(1.5%)
Rural-Urban Residence						
Metropolitan (SACTYPE 1)	161,712	(67.1%)	33,926	(70.5%)	30,315	(63.6%)
Small urban (SACTYPE 2–3)	51,674	(21.4%)	9858	(20.5%)	10,721	(22.5%)
Rural/remote (SACTYPE 4–7)	27,624	(11.5%)	4251	(8.8%)	5915	(12.4%)
Unknown	167	(0.1%)	103	(0.2%)	707	(1.5%)
Neighbourhood income quintile						
Q1 (lowest)	49,483	(20.5%)	14,010	(29.1%)	16,450	(34.5%)
Q2	47,486	(19.7%)	9947	(20.7%)	9885	(13.0%)
Q3	48,494	(20.1%)	8805	(18.3%)	7747	(16.3%)
Q4	49,671	(20.6%)	8134	(16.9%)	6590	(13.8%)
Q5 (highest)	43,770	(18.1%)	6704	(13.9%)	5109	(10.7%)
Missing	2273	(0.9%)	538	(1.1%)	1877	(3.9%)
Income assistance prescription drug coverage (plan C)	25,818	(10.7%)	16,394	(34.1%)	22,014	(46.2%)
Charlson-Deyo index (mean, SD)	0.8	± 1.6	0.9	± 1.6	1.0	± 1.8
Hospital admission in 2016/7						
MSUD hospital admission	7350	(3.0%)	9371	(19.5%)	16,279	(34.2%)
Involuntary hospitalisation	2845	(1.2%)	6335	(13.2%)	7837	(16.4%)

**Notes:** Cohorts were based on prevalence and typical severity of the disorders. Common mental disorders included depressive disorders, anxiety disorders, and post-traumatic stress disorder. Serious mental disorders comprised schizophrenia spectrum, and bipolar disorders. People treated for both a common and serious MD were grouped into the serious MD cohort. Substance use disorders included alcohol-related disorders, opioid-related disorders, cannabis-related disorders, stimulant-related disorders, and other substance use/abuse. People treated for both a MD (common and/or serious) and a SUD were grouped into the SUDs cohort

We observed that 5.9% of people treated for common MDs, 14.1% of people treated for serious MDs, and 29.1% of people treated for SUDs in 2016/7 had one or more MSUD ED visits in 2017/8 (Table 2). Within these cohorts, 29.4, 26.4, and 31.2% had a non-MSUD ED visit in 2017/8 among people with common MDs, serious MDs, and SUDs, respectively. As our study cohorts included people treated for MSUDs in 2016/7 (including services provided by primary care, other outpatient services, and in hospital), access to primary care was generally high, with only 1.2% of people with common MDs and 7.1% of people with serious MDs, and 8.8% of people with SUDs having no primary care visits. The three cohorts were similar with respect to number of primary care visits, with an average between 9.8 and 10.5 per person, per year across cohorts (Table 2); of these, between 3.2 and 3.7 visits were for MSUD services. Continuity of care was slightly lower for people treated for SUDs. Among people treated for SUDs they also had an additional 13.5 visits annually with a primary care provider related to opioid agonist therapy, on average. About a fifth (17.8%) of people with common MDs and 76.3% of people with serious MDs had one or more visits with a psychiatrist, whereas psychiatric care was much less common, at only 24.3%, among people with SUDs.

**Table 2:** Emergency department (ED) visits and outpatient service use, by cohort. N(%) except where indicated

	Common mental disorders *N* = 241,177 (71.6%)		Serious mental disorders *N* = 48,138 (14.3%)		Substance use disorders *N* = 47,658 (14.1%)	
Emergency department visits in 2017/8						
MSUD ED visits (mean, SD)	0.1	± 0.5	0.3	± 1.2	0.9	± 3.2
0	227,018	(94.1%)	41,373	(85.9%)	33,783	(70.9%)
1	10,084	(4.2%)	3594	(7.5%)	6378	(13.4%)
2–5	3798	(1.6%)	2763	(5.7%)	5668	(11.9%)
6–11	238	(0.1%)	330	(0.7%)	1242	(2.6%)
12+	39	(0.0%)	78	(0.2%)	587	(1.2%)
Other ED visits	70,836	(29.4%)	12,708	(26.4%)	14,848	(31.2%)
Outpatient service use 2016/7 or 365 days before first ED visit in 2017/8						
Primary care visits (mean, SD)	10.5	± 8.5	9.8	± 9.2	10.4	± 10.0
0 visits	2794	(1.2%)	3417	(7.1%)	4176	(8.8%)
1 visit	5565	(2.3%)	2475	(5.1%)	2905	(6.1%)
2 visits	10,618	(4.4%)	2735	(5.7%)	2904	(6.1%)
3+ visits	222,200	(92.1%)	39,511	(82.1%)	37,673	(79.0%)
MSUD primary care visits (mean, SD)	3.2	± 3.1	3.2	± 4.2	3.7	± 5.0
0 visits	18,521	(7.7%)	13,414	(27.9%)	13,537	(28.4%)
1 visit	49,340	(20.5%)	7862	(16.3%)	7288	(15.3%)
2 visits	54,878	(22.8%)	6683	(13.9%)	5714	(12.0%)
3+ visits	118,438	(49.1%)	20,179	(41.9%)	21,119	(44.3%)
Continuity of care index	5.8	± 3.4	5.5	± 3.7	4.3	± 3.5
Psychiatrist Visits (mean, SD)	1.0	± 4.1	5.5	± 7.8	1.5	± 4.7
0 visits	198,185	(82.2%)	11,442	(23.8%)	36,091	(75.7%)
1 visit	10,750	(4.5%)	3307	(6.9%)	3011	(6.3%)
2 visits	6597	(2.7%)	4231	(8.8%)	1684	(3.5%)
3+ visits	25,645	(10.6%)	29,158	(60.6%)	6872	(14.4%)

**Notes:** Common mental disorders included depressive disorders, anxiety disorders, and post-traumatic stress disorder. Serious mental disorders comprised schizophrenia spectrum, and bipolar disorders. People treated for both a common and serious MD were grouped into the serious MD cohort. Substance use disorders included alcohol-related disorders, opioid-related disorders, cannabis-related disorders, stimulant-related disorders, and other substance use abuse. People treated for both a MD (common and/or serious) and a SUD were grouped into the SUDs cohort

Associations between ED visits and outpatient service use differed by cohort (Table 3). In adjusted analysis, having 3+ primary care visits for any reasons showed different patterns. Among people with common and serious MDs it was associated with lower odds of any ED visit, but it was not associated with rate of ED visits. The reverse pattern was observed for people with SUDs. Having 3+ primary care visits was not associated with any ED visit but with a lower rate of ED visits. Having had one or more MSUD-related primary care visit was associated with lower odds of any ED visits among people treated for common MDs and SUDs but not among people with serious MDs. Continuity of primary care was consistently associated with both slightly lower odds of any ED visits and count of ED visits among all three cohorts (Table 3). Having had one or more outpatient psychiatrist visits was associated with lower odds of an ED visit among people treated for serious MDs and with lowers odds of both any ED visit and count of ED visits among people treated for SUDs (Table 3).

**Table 3:** Odds/rate ratios (95%CI) of mental disorder (MD) emergency department (ED) visits and outpatient service use

Common mental disorders	Unadjusted			Adjusted		
**Any MSUD ED visit**	**OR**	**95% CI** **Upper confidence interval**	**Lower confidence interval**	**OR**	**95% CI** **Upper confidence interval**	**Lower confidence interval**
1 primary care visit (vs. 0)	0.54	0.47	0.62	0.82	0.68	1.00
2 primary care visits (vs. 0)	0.36	0.32	0.41	0.84	0.70	1.02
3+ primary care visits (vs. 0)	0.34	0.31	0.38	0.70	0.59	0.83
1+ primary care visit related to MSUD (vs. 0)	0.53	0.50	0.56	0.80	0.75	0.87
Continuity of Care Index (range 0–10)	0.93	0.92	0.93	0.96	0.96	0.97
1+ psychiatrist visit (vs. 0)	1.69	1.62	1.76	0.98	0.93	1.03
**Count of MSUD ED visits**	**RR**	**95% CI**		**RR**	**95% CI**	
1 primary care visit (vs. 0)	0.71	0.54	0.93	0.85	0.64	1.13
2 primary care visits (vs. 0)	0.89	0.69	1.15	1.04	0.79	1.37
3+ primary care visits (vs. 0)	0.71	0.58	0.87	0.88	0.69	1.12
1+ primary care visit related to MSUD (vs. 0)	1.01	0.91	1.12	1.06	0.93	1.20
Continuity of Care Index (range 0–10)	0.98	0.97	0.99	0.97	0.96	0.99
1+ psychiatrist visit (vs. 0)	1.48	1.36	1.60	1.06	0.98	1.14
**Serious mental disorders**	**Unadjusted**			**Adjusted**		
**Any MSUD ED visit**	**OR**	**95% CI**		**OR**	**95% CI**	
1 primary care visit (vs. 0)	0.84	0.74	0.96	0.92	0.74	1.14
2 primary care visits (vs. 0)	0.72	0.63	0.82	1.10	0.87	1.37
3+ primary care visits (vs. 0)	0.61	0.56	0.67	0.75	0.62	0.89
1+ primary care visits related to MSUD (vs. 0)	0.94	0.88	0.99	1.09	0.99	1.20
Continuity of Care Index (range 0–10)	0.93	0.92	0.94	0.97	0.96	0.98
1+ psychiatrist visit (vs. 0)	1.12	1.05	1.19	0.78	0.72	0.85
**Count of MSUD ED visits**	**RR**	**95% CI**		**RR**	**95% CI**	
1 primary care visit (vs. 0)	0.89	0.70	1.13	0.92	0.77	1.10
2 primary care visits (vs. 0)	1.03	0.81	1.31	1.13	0.93	1.37
3+ primary care visits (vs. 0)	0.85	0.73	1.00	1.04	0.89	1.21
1+ primary care visit related to MSUD (vs. 0)	0.95	0.85	1.05	1.02	0.92	1.12
Continuity of Care Index (range 0–10)	0.97	0.96	0.98	0.98	0.97	1.00
1+ psychiatrist visit (vs. 0)	1.34	1.19	1.51	1.04	0.95	1.14
**Substance use disorders**	**Unadjusted**			**Adjusted**		
**Any MSUD ED visit**	**OR**	**95% CI**		**OR**	**95% CI**	
1 primary care visit (vs. 0)	0.81	0.74	0.90	1.01	0.88	1.17
2 primary care visits (vs. 0)	0.76	0.69	0.84	1.21	1.05	1.41
3+ primary care visits (vs. 0)	0.59	0.55	0.63	0.98	0.87	1.09
1+ primary care visits related to MSUD (vs. 0)	0.52	0.50	0.55	0.80	0.74	0.86
Continuity of Care Index (range 0–10)	0.87	0.87	0.88	0.93	0.92	0.94
1+ psychiatrist visit (vs. 0)	1.47	1.41	1.54	0.69	0.64	0.74
**Count of MSUD ED visits**	**RR**	**95% CI**		**RR**	**95% CI**	
1 primary care visit (vs. 0)	0.92	0.78	1.08	0.89	0.76	1.04
2 primary care visits (vs. 0)	1.00	0.85	1.18	0.97	0.82	1.14
3+ primary care visits (vs. 0)	0.84	0.76	0.93	0.78	0.69	0.88
1+ primary care visit related to MD (vs. 0)	1.16	1.07	1.25	1.22	1.11	1.34
Continuity of Care Index (range 0–10)	0.96	0.96	0.97	0.97	0.96	0.98
1+ psychiatrist visit (vs. 0)	1.30	1.21	1.40	0.86	0.79	0.93

Notes: Odds Ratios (OR) represent the association between primary care visits, continuity of care index, psychiatrist visits (predictors) and any ED visits (outcome).

Rate Ratios (RR) represent the association between primary care visits, continuity of care index, psychiatrist visits (predictors) and count of ED visits (outcome) among those with > 1 ED visits.

Adjusted models include all outpatient service use variables as well as individual characteristics displayed in Table 1

## Discussion

The characteristics of people treated for MSUDs in this large BC cohort mirror those described in other jurisdictions. Our findings indicate that fewer men than women were treated for MDs, especially for common MDs, while fewer women were treated for SUDS, reflecting gendered differences seen elsewhere [35, 49, 50]. That fewer men were treated for MD’s potentially signals men’s reticence to acknowledge and seek help for mental health concerns [51], and/or a preference for self-management [52]. Consistent with the well-established association between poverty and serious MDs [53] more people with serious MDs lived in low-income neighbourhoods and received prescription drug coverage associated with income assistance. Also unsurprisingly, people with serious MDs and SUDs had higher rates of both voluntary and involuntary hospitalizations, reflecting more complex needs overall.

Associations between outpatient service use and ED visits varied substantially across cohorts. Primary care may have a particularly important role to play among people with common MDs, for whom having 3+ primary care visits for any reason, and one or more MSUD-related primary care visits, were associated with lower odds of any ED visit. This is consistent with primary care providers’ capacity and expertise in managing common disorders, compared to serious MDs and SUDs which generally require greater specialization [39–42].

Among people with serious MDs having 3+ primary care visits for any reason and one or more psychiatry visits (but not one or more primary care visits for MSUDs) were associated with lower odds of any ED visit. Similarly, among people with SUDs having one or more outpatient psychiatrist visit was associated with both lower odds of any ED visit and a lower count of ED visits. Findings point to the importance of specialized expertise in managing more serious conditions [41, 42] and mirror associations observed elsewhere [14, 15, 20, 23–25].

Only a quarter of people with SUDs had an outpatient visit with a psychiatrist, despite the fact that over half had co-occurring MDs. That so few people in the SUD cohort saw a psychiatrist is alarming, though not unexpected as concurrent MSUDs are associated with higher levels of unmet need [54]. This trend may indicate concurrent MSUDs are not receiving concurrent, coordinated, or integrated care despite evidence that integrative rather than sequential treatment is best for people with concurrent disorders [55–57].

Continuity of primary care was associated with somewhat lower ED use (both odds and rate ratios) across all three cohorts, as has been found in other studies [7, 24, 25, 58, 59]. However, it is also possible that some of the association between continuity of care and ED visits reflects unmeasured confounding. Though we control for measures of income, it is possible that people with more resources (e.g., social support) or stability (e.g., residing in the same location) are better able to consistently visit the same primary care provider, and are also less likely to seek care in EDs [60]. Similarly, while psychiatry visits were associated with reduced ED visits for serious MDs and SUDs, we cannot determine if these visits helped to stabilize individuals and avert them from the ED or if psychiatry visits signal preceding stability (e.g., organizational skills needed to navigate access).

Findings reflect that service remains fragmented and collaborative models are not widely implemented in BC [57]. Roughly a quarter of people with serious MDs and three quarters of people with SUDs had no psychiatry visits and roughly a quarter also had no visits with a primary care provider related to MDs. This signals barriers to accessing integrated care for people with serious MDs [61, 62] and a lack of capacity for collaborative care between primary care and psychiatry services [39, 40, 42]. Collaborative and integrative care models may better support primary care providers treating people with serious MDs and SUDs [41, 42], which is especially important given the relative lack of access to outpatient psychiatric, and psychological services in Canada [62, 63].

### Strengths and limitations

Our study includes all people treated for MSUDs within a publicly funded health insurance system. The availability of province-wide linked administrative data permits the analysis of associations between outpatient services use and ED visits within large, population-based cohorts of people treated for mental disorders. Use of population-based data make it possible to examine associations with any ED visits, not just frequency of ED visits within cohorts identified in ED, as has been typical in other studies (e.g., [49]). We expect findings may apply in other settings with a gatekeeping model of primary care, and where there is limited implementation of collaborative or integrated models of mental health service delivery within primary care.

As our analysis is restricted to people who were already accessing publicly funded services, it does not capture people with untreated conditions, people seeking informal and/or private supports, people receiving psychotherapy or counselling in mental health community centres, or people struggling to access care for whom an ED visit was their first-ever MSUD service use (approximately 25% of ED contacts in BC) [64]. Findings are also limited by the fact that data were not collected for research. MSUD severity is an important consideration for treatment and health services provisions [65, 66]; while hospitalization can serve as a proxy for severity, administrative data does not otherwise capture disorder severity. We grouped disorders based on the most common or typical severity of the disorder, alongside prevalence, but some people may have been misclassified. We also collapsed people with MD and SUD into the SUD cohort, future research should disentangle these two groups. In addition, individuals with MDs have diverse needs that can benefit from support from a variety of providers (e.g., nurses, psychologists, social workers, occupational therapists); however, interactions with these providers cannot be observed in our data. Future research examining the impact of other supports and services (e.g., nursing, psychological services, social work, peer support, occupational therapy, etc.) on ED visits for MDs is needed. Finally, our data do not measure access to housing, social, or economic supports that have consistent associations with ED visits [18, 19, 67, 68].

## Conclusions

Associations between outpatient service use and ED visits varied across cohorts. While access to continuous primary care is important for everyone, use of outpatient psychiatry services was associated with lower ED use among people with serious MDs and particularly among people with SUDs. Findings point to the urgent need for expanded access to specialist mental health services, as well as collaborative and integrated care models that can better support primary care providers treating people with MSUDs.

### Supplementary Information

**Additional file 1.**

## Data Availability

The data that support the findings of this study are available from Population Data BC following submission of a data access request (https://www.popdata.bc.ca/data_access). De-identified data were provided to the research team. We are not permitted to share the research extract used in this analysis with other researchers.

## Appendix

**Table 4:** Disorder groupings and associated diagnosis codes in British Columbia administrative data

	ICD-9 codes (MSP claims)	ICD-10 codes (Hospital Discharge Data)	NACRS Codes
Common mental disorders			
Depressive disorders	311	F32, F33, F34.1	F329
Anxiety disorders	300	F40, F41	F419
Anxiety/depression (code unique to MSP)	50B	n/a	n/a
Post-Traumatic Stress Disorder	308, 309	F43	n/a
Serious mental disorders			
Bipolar and related disorders	296	F31, F34 (excluding F34.1), F38, F39	n/a
Schizophrenia spectrum and other psychotic disorders	295, 297, 298	F20, F21, F22, F23, F24, F25, F28, F29	F209, F239
Substance use disorders			
Alcohol-related disorders	291, 303	F10	F100, F103, T510
Opioid-related disorders	292, 304, 305	F11	F119, T401
Cannabis-related disorders	292, 304, 305	F12	F129, T407
Stimulant-related disorders	292, 304, 305	F14	F149, F159, T405
Other substance use abuse	292, 304, 305	F13, F16, F17, F18, F19	F139, F169, T409, T406, F180, F199, T424, T439

**Table 5:** List of fee codes included in identification of ED visits using Medical Services Plan Payment data

FEE ITEM	DESCRIPTION
1811	LEVEL I EMERGENCY CARE - DAY
1812	01812 LEVEL II EMERGENCY CARE - DAY
1813	01813 LEVEL III EMERGENCY CARE - DAY
1821	01821 LEVEL I EMERGENCY CARE - EVENING
1822	01822 LEVEL II EMERGENCY CARE - EVENING
1823	01823 LEVEL III EMERGENCY CARE - EVENING
1831	01831 LEVEL I EMERGENCY CARE - NIGHT
1832	01832 LEVEL II EMERGENCY CARE - NIGHT
1833	01833 LEVEL III EMERGENCY CARE - NIGHT
1841	01841 LEVEL I EMERGENCY CARE - SAT, SUN, OR STAT HOL
1842	01841 LEVEL I EMERGENCY CARE - SAT, SUN, OR STAT HOL
1843	01841 LEVEL I EMERGENCY CARE - SAT, SUN, OR STAT HOL
96,801	96,801 APB-LEVEL I EMERGENCY CARE DAY
96,802	96,802 APB - LEVEL 2 EMERGENCY CARE - DAY
96,803	96,803 APB - LEVEL 3 EMERGENCY CARE - DAY
96,804	96,804 APB- LEVEL 4 EMERGENCY CARE - DAY
96,805	96,805 APB - LEVEL 5 EMERGENCY CARE - DAY
96,811	96,811 APB-LEVEL I EMERGENCY CARE - EVENING
96,812	96,812 APB - LEVEL 2 EMERGENCY CARE - EVENING
96,813	96,813 APP - LEVEL 3 EMERGENCY CARE - EVENING
96,814	96,814 APB - LEVEL 4 EMERGENCY CARE - EVENING
96,815	96,815 APB - LEVEL 5 EMERGENCY CARE - EVENING
96,821	96,821 APB - LEVEL 1 EMERGENCY CARE - NIGHT
96,822	96,822 APB - LEVEL 2 EMERGENCY CARE - NIGHT
96,823	96,823 APB -LEVEL 3 EMERGENCY CARE - NIGHT
96,824	96,824 APB - LEVEL 4 EMERGENCY CARE - NIGHT
96,825	96,825 APB - LEVEL 5 EMERGENCY CARE - NIGHT
36,347	36,347 NP - VISIT, EMERGENCY (BETWEEN 0800 AND 1800 HRS)
36,440	36,440 NP - SIMPLE/FASTRACK VISIT IN EMERGENCY (AGE 50–59)
36,441	36,441 NP - EMERGENCY DEPARTMENT VISIT (AGE 50–59)
36,447	36,447 NP - SIMPLE/FASTRACK VISIT IN EMERGENCY (AGE 2–19)
36,448	36,448 NP - EMERGENCY DEPARTMENT VISIT (AGE 2–19)
36,601	36,601 NP - SIMPLE/FASTRACK VISIT IN EMERGENCY (AGE 0–1)
36,602	36,602 NP - SIMPLE/FASTRACK VISIT IN EMERGENCY (AGE 2–59)
36,603	36,603 NP - SIMPLE/FASTRACK VISIT IN EMERGENCY (AGE 60–69)
36,604	36,604 NP - SIMPLE/FASTRACK VISIT IN EMERGENCY (AGE 70–79)
36,605	36,605 NP - SIMPLE/FASTRACK VISIT IN EMERGENCY (AGE 80+)
36,606	36,606 NP - VISIT IN EMERGENCY DEPARTMENT (AGE 0–1)
36,607	36,607 NP - VISIT IN EMERGENCY DEPARTMENT (AGE 2–59)
36,608	NP - VISIT IN EMERGENCY DEPARTMENT (AGE 60–69)
36,609	NP - VISIT IN EMERGENCY DEPARTMENT (AGE 70–79)
36,610	NP - VISIT IN EMERGENCY DEPARTMENT (AGE 80+)

## Abbreviations

BC: British Columbia
COCI: Continuity of Care Index
ED: Emergency Department
MD: Mental Disorder
MSP: Medical Service Plan
MSUD: Mental and Substance Use Disorder
NACRS: National Ambulatory Care Reporting System
PTSD: Posttraumatic Stress Disorder
SUD: Substance Use Disorder
