# Supplementary material for: The relationship between outpatient service use and emergency department visits among people treated for mental and substance use disorders: analysis of population-based administrative data in British Columbia, Canada
Source: BMC Health Serv Res. 2022 Apr 11;22:477. doi: 10.1186/s12913-022-07759-z (PMC8996395; doi:10.1186/s12913-022-07759-z)
Supplement: Supplementary file 1 — Additional file 1. [file 12913_2022_7759_MOESM1_ESM.docx]

**Appendix 1**. Disorder groupings and associated diagnosis codes in British Columbia administrative data

|  | ICD-9 codes  (MSP claims) | ICD-10 codes  (Hospital Discharge Data) | NACRS Codes |
| --- | --- | --- | --- |
| **Common mental disorders** |  |  |  |
| Depressive disorders | 311 | F32, F33, F34.1 | F329 |
| Anxiety disorders | 300 | F40, F41 | F419 |
| Anxiety/depression (code unique to MSP) | 50B | n/a | n/a |
| Post-Traumatic Stress Disorder | 308, 309 | F43 | n/a |
| **Serious mental disorders** |  |  |  |
| Bipolar and related disorders | 296 | F31, F34 (excluding F34.1), F38, F39 | n/a |
| Schizophrenia spectrum and other psychotic disorders | 295, 297, 298 | F20, F21, F22, F23, F24, F25, F28, F29 | F209, F239 |
| **Substance use disorders** |  |  |  |
| Alcohol-related disorders | 291, 303 | F10 | F100, F103, T510 |
| Opioid-related disorders | 292, 304, 305 | F11 | F119, T401 |
| Cannabis-related disorders | 292, 304, 305 | F12 | F129, T407 |
| Stimulant-related disorders | 292, 304, 305 | F14 | F149, F159, T405 |
| Other substance use abuse | 292, 304, 305 | F13, F16, F17, F18, F19 | F139, F169, T409, T406, F180, F199, T424, T439 |

**Appendix 2.** List of fee codes included in identification of ED visits using Medical Services Plan Payment data

| **FEE ITEM** | **DESCRIPTION** |
| --- | --- |
| 1811 | LEVEL I EMERGENCY CARE - DAY |
| 1812 | 01812 LEVEL II EMERGENCY CARE - DAY |
| 1813 | 01813 LEVEL III EMERGENCY CARE - DAY |
| 1821 | 01821 LEVEL I EMERGENCY CARE - EVENING |
| 1822 | 01822 LEVEL II EMERGENCY CARE - EVENING |
| 1823 | 01823 LEVEL III EMERGENCY CARE - EVENING |
| 1831 | 01831 LEVEL I EMERGENCY CARE - NIGHT |
| 1832 | 01832 LEVEL II EMERGENCY CARE - NIGHT |
| 1833 | 01833 LEVEL III EMERGENCY CARE - NIGHT |
| 1841 | 01841 LEVEL I EMERGENCY CARE - SAT, SUN, OR STAT HOL |
| 1842 | 01841 LEVEL I EMERGENCY CARE - SAT, SUN, OR STAT HOL |
| 1843 | 01841 LEVEL I EMERGENCY CARE - SAT, SUN, OR STAT HOL |
| 96801 | 96801 APB-LEVEL I EMERGENCY CARE DAY |
| 96802 | 96802 APB - LEVEL 2 EMERGENCY CARE - DAY |
| 96803 | 96803 APB - LEVEL 3 EMERGENCY CARE - DAY |
| 96804 | 96804 APB- LEVEL 4 EMERGENCY CARE - DAY |
| 96805 | 96805 APB - LEVEL 5 EMERGENCY CARE - DAY |
| 96811 | 96811 APB-LEVEL I EMERGENCY CARE - EVENING |
| 96812 | 96812 APB - LEVEL 2 EMERGENCY CARE - EVENING |
| 96813 | 96813 APP - LEVEL 3 EMERGENCY CARE - EVENING |
| 96814 | 96814 APB - LEVEL 4 EMERGENCY CARE - EVENING |
| 96815 | 96815 APB - LEVEL 5 EMERGENCY CARE - EVENING |
| 96821 | 96821 APB - LEVEL 1 EMERGENCY CARE - NIGHT |
| 96822 | 96822 APB - LEVEL 2 EMERGENCY CARE - NIGHT |
| 96823 | 96823 APB -LEVEL 3 EMERGENCY CARE - NIGHT |
| 96824 | 96824 APB - LEVEL 4 EMERGENCY CARE - NIGHT |
| 96825 | 96825 APB - LEVEL 5 EMERGENCY CARE - NIGHT |
| 36347 | 36347 NP - VISIT, EMERGENCY (BETWEEN 0800 AND 1800 HRS) |
| 36440 | 36440 NP - SIMPLE/FASTRACK VISIT IN EMERGENCY (AGE 50-59) |
| 36441 | 36441 NP - EMERGENCY DEPARTMENT VISIT (AGE 50-59) |
| 36447 | 36447 NP - SIMPLE/FASTRACK VISIT IN EMERGENCY (AGE 2-19) |
| 36448 | 36448 NP - EMERGENCY DEPARTMENT VISIT (AGE 2-19) |
| 36601 | 36601 NP - SIMPLE/FASTRACK VISIT IN EMERGENCY (AGE 0-1) |
| 36602 | 36602 NP - SIMPLE/FASTRACK VISIT IN EMERGENCY (AGE 2-59) |
| 36603 | 36603 NP - SIMPLE/FASTRACK VISIT IN EMERGENCY (AGE 60-69) |
| 36604 | 36604 NP - SIMPLE/FASTRACK VISIT IN EMERGENCY (AGE 70-79) |
| 36605 | 36605 NP - SIMPLE/FASTRACK VISIT IN EMERGENCY (AGE 80+) |
| 36606 | 36606 NP - VISIT IN EMERGENCY DEPARTMENT (AGE 0-1) |
| 36607 | 36607 NP - VISIT IN EMERGENCY DEPARTMENT (AGE 2-59) |
| 36608 | NP - VISIT IN EMERGENCY DEPARTMENT (AGE 60-69) |
| 36609 | NP - VISIT IN EMERGENCY DEPARTMENT (AGE 70-79) |
| 36610 | NP - VISIT IN EMERGENCY DEPARTMENT (AGE 80+) |
